# NTRK amplification occurs frequently in pan-TRK immunopositive dedifferentiated liposarcomas

**DOI:** 10.3389/pore.2024.1611993

**Published:** 2025-01-07

**Authors:** Zoltán Lippai, Gergő Papp, Károly Szuhai, Johanna Sápi, Katalin Dezső, Zoltán Sápi

**Affiliations:** ^1^ Department of Pathology and Experimental Cancer Research, Semmelweis University, Budapest, Hungary; ^2^ Department of Cell and Chemical Biology, Leiden University Medical Center, Leiden, Netherlands; ^3^ John von Neumann Faculty of Informatics, Physiological Controls Research Center, Obuda University, Budapest, Hungary

**Keywords:** soft tissue sarcoma, dedifferentiated liposarcoma, gene rearrangement, gene amplification, NTRK

## Abstract

The *neurotrophic tyrosine kinase receptor* (*NTRK*) gene family is of rising importance as their fusions are oncogenic, and specific target drugs are available to inhibit the chimera proteins. Pan-TRK antibody, which shows the overexpression of the *NTRK1-2-3* genes, is a useful tool to detect tumors with or without *NTRK* gene alterations, due to high negative predictive value. Though it is well known that pan-TRK immunopositivity is usually not connected to *NTRK* fusion, the role of other possible genetic alterations is under-researched. In our previous work, we found 3 *NTRK1* amplified cases out of 6 cases with recurrent *NTRK1* tyrosine kinase domain mutation pair, so we extended our investigation to a larger series to estimate amplification frequency. Pan-TRK immunopositivity was seen in 76 of the 132 dedifferentiated liposarcomas cases, followed by *NTRK1-2-3* break-apart FISH tests in 76 pan-TRK positive cases to detect oncogenic fusions or other copy number alterations of these genes. None of the pan-TRK immunopositive dedifferentiated liposarcomas showed absolutely certain sign of fusion, however, 18 (28%) cases showed amplification of one of the genes, 13 had polysomy, 34 were normal, 11 were not evaluable. The extent of pan-TRK immunoreaction showed a positive correlation (p = 0.002) with the *NTRK* status found by FISH. Analyzing publicly available data from large series of 265 liposarcoma samples consisting of both well-differentiated and dedifferentiated liposarcoma case, 23 (8.6%) cases showed a mutual exclusive amplification of the *NTRK* genomic loci in a non-preselected, independent patient population indicating that our findings are presented in other cohorts. Our results underline the so far not revealed frequent occurrence of *NTRK* amplifications which might be important in the TRK inhibition therapy.

## Introduction

### Background

The role of *NTRK* fusion and overexpression of the TRK receptor has great clinical relevance in soft tissue sarcomas as targeted therapy option is available for those patients [1–4]. Extensive investigation has been performed to identify novel entities or tumor subtypes with the involvement of the *NTRK* genes.

### *NTRK* gene and pan-TRK immunohistochemistry

The *neurotrophic tyrosine kinase receptor* (*NTRK*) gene family consists of three genes: *NTRK1*, *NTRK2* and *NTRK3* [5]. These genes are localized on different chromosomes, respectively: 1q23.1, 9q21.33, and 15q25.3 [5]. These genes encode three tyrosine kinase receptors (TRKA, TRKB and TRKC), that play a role in the regulation of apoptosis, survival, differentiation and proliferation in several cell types through well-known downstream signaling pathways, including PLCγ/PKC, MAPK/ERK and PI3K/AKT [5]. The tyrosine kinase receptors consist of extracellular, ligand-binding domain, transmembrane domain and intracellular, tyrosine kinase domain [5, 6]. The most important gene alterations affecting the *NTRK* gene family are fusions [6, 7]. *NTRK* fusions are characteristic in some rare tumors, like infantile fibrosarcoma [8], and congenital mesoblastic nephroma [9], but can occur in common malignancies as well, like lung cancer [10], colorectal cancer [11], melanoma [12], though less frequently. However, the fusions are the most investigated and best known, other gene alterations can occur in the *NTRK* genes. Recently, oncogenic point mutations within the extracellular, transmembrane and tyrosine kinase domains as well were identified in haematological malignancies [13, 14]. Furthermore, a deletion in the extracellular domain of TRKA showed oncogene capability in acute myeloid leukemia [15]. Also, a TRKA splice variant promoted tumorigenic cell behavior [16]. Although, *NTRK1* amplification occurs in 8% of breast cancers [17], and several non-fusion *NTRK* alterations have been described in 14% of adult and pediatric tumors as well, their role in oncogenesis is yet to be explored [18, 19]. The *NTRK* gene family is at the center of attention nowadays, due to the highly effective, selective TRK inhibitors [20]. The US Food and Drug Administration approved the usage of specific TRK inhibitors, larotrectinib [1] and entrectinib [2], in case of any malignancy with *NTRK* fusion, regardless of the exact entity. Due to acquired resistance mutations within the tyrosine kinase domain, second-generation TRK inhibitors, repotrectinib [3] and selitrectinib [4], were developed.

Pan-TRK immunohistochemistry, which indicates the presence of TRKA and/or TRKB and/or TRKC protein(s), is a valuable tool to identify tumors without *NTRK* gene rearrangements, as it has a high negative predictive value [21]. Pan-TRK immunopositivity can be detected in some soft tissue sarcomas showing myogenic phenotype or myogenic differentiation, without *NTRK* fusion [22, 23].

In our previous work we investigated 131 dedifferentiated liposarcoma (DDLPS) cases and found 75 pan-TRK immunohistochemically positive cases [24]. Among the pan-TRK positive cases we found 6 cases with recurrent *NTRK1* c.1810C>T (p.H604Y) and c.1838G>T (p.G613V) tyrosine kinase domain mutation pair and interestingly 3 of this 6 cases showed *NTRK1* amplification. This surprisingly high frequency prompted us to extend our investigation and to estimate the real amplification frequency in this 75 cases. One additional pan-TRK positive case was included, so the total number of investigated cases were 76.

Our hypothesis was to investigate the relation between pan-TRK immunopositivity and possible genetic alterations by using *NTRK1/2/3* locus specific break apart probe set in order to estimate the frequency of genomic rearrangement and/or regional or overall copy number alterations related to any of these three regions.

## Materials and methods

### Case selection

We analyzed 76 individual cases of dedifferentiated liposarcoma with myogenic heterologous differentiation and pan-TRK immunopositivity from our institutional and consultation archives (diagnosed between 2014 and 2024) in this study. The diagnosis was based on clinical features, characteristic morphology and immunohistochemical and/or molecular examination of *MDM2* and/or *CDK4* overexpression or amplification. Previously executed immunohistochemistry with desmin and alpha smooth muscle actin antibody were used to show myogenic heterologous differentiation, and pan-TRK antibody was used to detect *NTRK1-2-3* overexpression. This study was approved by the Semmelweis University Regional and Institutional Committee of Science and Research Ethics (TUKEB 155/2012).

### Immunohistochemistry (IHC)

Immunohistochemistry was performed on deparaffinized, rehydrated whole slide formalin-fixed, paraffin-embedded (FFPE) block sections with the Bond-Max automated staining system (Leica Biosystems, Wetzlar, Germany), after applying antibody-specific epitope retrieval techniques, using the following antibody: pan-TRK (ready-to-use, EPR17341, Ventana). The intensity and extent of immunoreactivity were evaluated by two soft tissue pathologists (ZS and KD). Tumor samples were considered positive with pan-TRK antibody if >1% of tumor cells exhibited immunostaining at any intensity above the background. Staining intensities of tumor cells were graded as no staining (0), very weak (1), weak (2), moderate (3), strong (4) or very strong (5) and numbers of stained tumor cells as the extent of staining were graded as 0% (0), 1%–19% (1), 20%–39% (2) 40%–59% (3), 60%–79% (4), 80%–100% (5).

### Fluorescence *in situ* hybridization (FISH)

FISH was performed on interphase nuclei from 3 µm sections of FFPE blocks using the ZytoLight SPEC Dual Color Break Apart Probes of the *NTRK1/2/3* genes (ZytoVision, Bremerhaven, Germany). The slides were analyzed by assessing a minimum of 100 cells each in a tumor region of interest. Rearrangement of the *NTRK* genes was defined as a minimum of 20% tumor cells with split signals (classical pattern) of the corresponding *NTRK* probes. Polysomy or copy number gain was defined by an average copy number of all *NTRK* (1/2/3) signals ≥3. Amplification was defined if a *NTRK* gene showed at least twice in number of signals compared to the average of the other two *NTRK* gene type. In other words, the different *NTRK*s (locating on different chromosomes) served as internal controls to assess whether there is a polysomy or either of them is amplified. Because we used break-apart *NTRK* probes, we could distinguish “whole” amplification (the whole gene was affected) or “partial” amplification (either the 3′ end/red signal or the 5′ end/green signal was amplified). FISH signals were scored by one pathologist (ZS) and one biologist (GP).

### Next-generation sequencing (NGS)

Targeted DNA and RNA libraries were prepared according to the TruSight Tumor 170 reference guide (Illumina, San Diego, CA, United States). The TruSight Tumor 170 kit included the analysis of 151 genes for insertion, deletion, single nucleotide variant, copy number variation and 55 genes for fusions and splice variants and 59 genes for amplification. Genomic DNA and total RNA were isolated from formalin-fixed, paraffin-embedded specimens using the QIAamp^®^ DNA FFPE Tissue Kit (Qiagen) and the High Pure FFPET RNA Isolation Kit (Roche Diagnostics, Mannheim, Germany). Estimations of tumor cell percentages of the samples were performed by histopathological examinations prior to the isolation processes. DNA and RNA concentrations were measured using the Qubit™ dsDNA HS Assay and Qubit™ RNA HS Assay Kits (Thermo Fisher Scientific) on a Qubit™ 4 Fluorometer. 120 ng DNA in 52 μL volume was sheared (200 cycles, peak power: 75 W, duty factor: 10, treatment time: 510 s, at 7°C setpoint) using a Covaris M220 Focused-ultrasonicator (Covaris, Woburn, United States). 80 ng of DNase digested RNA was used for library preparation of each sample The size of double-stranded DNA fragments and RNA molecules was confirmed after shearing using a Tapestation 4,200 (Agilent, Cheshire, UK). Library preparation workflow of Illumina TruSight Tumor 170 assay was performed according to the manufacturer’s protocol. The final libraries were paired-end sequenced at a 2 × 101 bp read length, using Illumina NextSeq 1000/2000 P3 (200 cycles) reagent kits on a NextSeq 2000 platform. Bioinformatic analysis was performed using TruSight Tumor 170 v2.0.2 Local App (Illumina, San Diego, CA, United States). For further clinical interpretation, we used Genoox’s Franklin web-based analysis tool, which applies variant filtering and further annotations: pathogenicity scores, population-frequency, protein structure predictions, relevant clinical guidelines and therapeutic options for variants.

### *In silico* analysis

Publicly available data sets from TCGA and cBioPortal were analysed for the presence of structural variants involving *NTRK1/2/3* gene loci. From three studies analyzing soft tissue sarcomas, well-differentiated and dedifferentiated liposarcoma cases were further analyzed to interrogate the frequencies of *NTRK1/2/3* genes and *MDM2* and *CDK4* genes. From the 2551 cases presented in these three studies 265 unique well-differentiated or dedifferentiated liposarcoma samples were included for the analysis using cBioPortal [25–29].

### Statistical analysis

*NTRK* gene family statuses by FISH (normal, polyploidy and amplification) were investigated based on pan-TRK immunohistochemistry (extent and intensity values). In order to compare three samples, one can use ANOVA test; however, in this case, there are two assumptions to be justified: 1. Normality of residuals (the errors used for the estimation of the error terms are normally distributed), 2. Homogeneity of variance (the level of variance for a particular variable is constant across the sample). For testing normality, we used one-sample Kolmogorov-Smirnov normal test; and Levene Statistic was used to test homogeneity of variances. Finally, to compare normal, polysomic and amplification groups, parametric one-way ANOVA and non-parametric Kruskal-Wallis tests were run.

## Results

**FIGURE 1:**
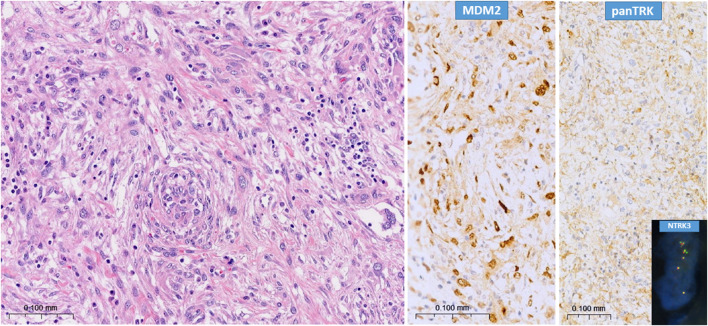
Typical picture of dedifferentiated liposarcoma (H&E) with characteristic strong MDM2 nuclear immunopositivity and strong pan-TRK cytoplasmic positivity. Insert shows *NTRK3* amplification (break-apart probe), case N°113.

We included 76 pan-TRK immunpositive dedifferentiated liposarcoma cases in our investigation. The diagnosis of dedifferentiated liposarcoma was based on histology and MDM2 and/or CDK4 immunohistochemistry findings (Figure 1). In cases with unequivocal immunohistochemistry results, FISH examinations were executed with *MDM2* and/or *CDK4* probes. The male-female ratio is 1.24 (42:34). The most common site for dedifferentiated liposarcoma in our setting was retroperitoneum (42%). Basic clinical data is available in an article of our previous study [24].

**FIGURE 2:**
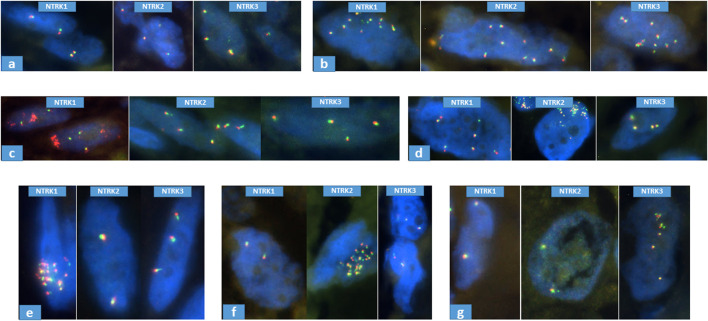
Representative samples of *NTRK* FISH results. **(A)**: normal, case N°20; **(B)**: polysomy, case N°85; **(C)**: partial amplification of *NTRK1* (3′), case N°7; **(D)**: partial amplification of *NTRK2* (5′), case N°115; **(E)**: whole amplification of *NTRK1*, case N°84; **(F)**: whole amplification of *NTRK2*, case N°11; **(G)**: whole amplification of *NTRK3*, case N°113.

With *NTRK1-2-3* FISH probes, among the 76 pan-TRK immunopositive cases, 34 proved to be normal as no copy number gain was evinced in the *NTRK* gene family. 13 cases proved to have polysomy because of the involvement of all three *NTRK* genes, which are localized on different chromosomes, showed increased number of signals. Mutually exclusive amplification pattern was seen in 18 cases (*NTRK1*: 9; *NTRK2*: 5; *NTRK3*: 4). 5 of these showed partial amplification since only the 3′ or the 5′ region of the given gene was amplified, while the other 13 cases showed the amplification of the whole sampled locus, as both, co-localised signals of the tested gene locus showed an increased copy number (Figure 2; Table 1). No classical *NTRK* fusion was detected by FISH. The partial amplification pattern is suggestive of fusion with unknown partners. To test possible fusions, we performed NGS (TruSight Tumor 170 kit) in one case of partial amplification, but no fusion was detected. Unfortunately, 11 cases were not evaluable in regard to *NTRK* gene family status due to FISH technical issues or insufficient amount of tumor material (Figures 1, 2; Table 1; Supplementary Table S1).

**TABLE 1:** *NTRK1-2-3* break-apart FISH results of amplified cases.

Patient ID	*NTRK* gene	FISH result	Copy number	Comment	Interpretation	Affected gene
7	*NTRK1*	extra red	green sign 3.8; red sign >10	*NTRK1* 3′ region amplification	amplification	*NTRK1*
	*NTRK2*	negative	4			
	*NTRK3*	negative	3			
8	*NTRK1*	negative	3.1		amplification	*NTRK2*
	*NTRK2*	negative	6.5	occasionally >10 sign		
	*NTRK3*	negative	3.2			
11	*NTRK1*	negative	2.2		amplification	*NTRK2*
	*NTRK2*	negative	7.4	occasionally >10 sign		
	*NTRK3*	negative	2.2			
23	*NTRK1*	negative	5.5	occasionally >10 sign	amplification	*NTRK1*
	*NTRK2*	negative	2.1			
	*NTRK3*	negative	2.4			
44	*NTRK1*	negative	2		amplification	*NTRK3*
	*NTRK2*	negative	2			
	*NTRK3*	negative	5.2			
123	*NTRK1*	negative	3.1		amplification	*NTRK3*
	*NTRK2*	negative	3.1			
	*NTRK3*	extra green	green sign 6.2; red sign 2.9	*NTRK3* 5′ region amplification		
129	*NTRK1*	negative	2.2		amplification	*NTRK2*
	*NTRK2*	negative	5.8			
	*NTRK3*	negative	3.1			
115	*NTRK1*	negative	8.2		amplification	*NTRK2*
	*NTRK2*	extra green	green sign >10; red sign ∼8	*NTRK2* 5′ region amplification		
	*NTRK3*	negative	7.5			
112	*NTRK1*	extra red	green sign 2.4; red sign >10	*NTRK1* 3′ region amplification	amplification	*NTRK1*
	*NTRK2*	negative	3.2			
	*NTRK3*	negative	4.8			
113	*NTRK1*	negative	3.5		amplification	*NTRK3*
	*NTRK2*	negative	3.6			
	*NTRK3*	negative	>10	whole *NTRK3* region amplification		
121	*NTRK1*	negative	2.2		amplification	*NTRK2*
	*NTRK2*	extra red	green sign 2; red sign >10	*NTRK2* 3′ region amplification		
	*NTRK3*	negative	2.7			
82	*NTRK1*	negative	2.5		amplification	*NTRK3*
	*NTRK2*	negative	2			
	*NTRK3*	negative	>10	whole *NTRK3* region amplification		
86	*NTRK1*	negative	9.5	whole *NTRK1* region amplification	amplification	*NTRK1*
	*NTRK2*	negative	3.2			
	*NTRK3*	negative	3.1			
51	*NTRK1*	negative	>10	whole *NTRK1* region amplification	amplification	*NTRK1*
	*NTRK2*	negative	3.4			
	*NTRK3*	negative	3.2			
132	*NTRK1*	negative	>10	whole *NTRK1* region amplification	amplification	*NTRK1*
	*NTRK2*	negative	2	no fusion by NGS		
	*NTRK3*	negative	2.2			
84	*NTRK1*	negative	>10	whole *NTRK1* region amplification	amplification	*NTRK1*
	*NTRK2*	negative	3.8	no fusion by NGS		
	*NTRK3*	negative	3.5			
88	*NTRK1*	negative	8.5	whole *NTRK1* region amplification	amplification	*NTRK1*
	*NTRK2*	negative	2	no fusion by NGS		
	*NTRK3*	negative	2			
125	*NTRK1*	negative	>10	whole *NTRK1* region amplification	amplification	*NTRK1*
	*NTRK2*	negative	2	no fusion by NGS		
	*NTRK3*	negative	2.5			

The intensity and the extent of pan-TRK immunoreactions varied over a wide range (Table 2; Supplementary Table S2; Supplementary Table S3).

**TABLE 2:** Detailed pan-TRK immunohistochemistry results of dedifferentiated liposarcoma cases with different *NTRK* gene family status.

*NTRK* gene family status by FISH	Pan-TRK immunohistochemistry		
	Score	Extent	Intensity
normal (n = 34)	1	13	28
	2	6	1
	3	5	4
	4	3	1
	5	7	0
	average	2.56	1.35
polysomy (n = 13)	1	2	8
	2	1	2
	3	2	2
	4	2	0
	5	6	1
	average	3.69	1.77
amplification (n = 18)	1	3	10
	2	0	3
	3	1	3
	4	1	0
	5	13	2
	average	4.17	1.94

265 samples consisting of 38 well-differentiated and 227 dedifferentiated liposarcoma cases were retrieved from cBioPortal. Amplification of the *NTRK1/2/3* genes were seen in 23/265 (8.6%) of the reported cases without preselection using pan-TRK immunohistochemistry for *NTRK1/2/3*. From these, 9/265 (3.3%), 11/265 (4.1%) and 3/265 (1.1%) cases showed amplification of the *NTRK1*, *NTRK2* and *NTRK3* genes, respectively. In all cases a mutually exclusive amplification pattern was observed. Only two cases (1 *NTRK1* and 1 *NTRK3*) with amplification were seen in well-differentiated liposarcoma samples. Single nucleotide variants (SNVs) of the *NTRK1/2/3* genes were reported in three cases, *NTRK1* G619R*, NTRK2* S180G and *NTRK3* D167H. Intriguingly, the *NTRK1* G619R was in a case with *NTRK2* amplification, and the *NTRK3* D167H was in a case with *NTRK1* amplification.

### Statistics for FISH and immunohistochemistry results

The statistical analysis showed that in all groups (normal, polysomy and amplification) the assumption of normality of the residues is not met, neither in terms of extent nor in terms of intensity. The homogeneity of variance was found to be fulfilled in terms of the extent. Since simulation studies using a variety of non-normal distributions have shown that the false positive rate is not affected very much by the violation of the normality assumption [30, 31], we used one-way ANOVA to investigate extent. It resulted in p = 0.002, which means that the groups are significantly different, moreover *post hoc* test showed the following p values: p = 0.075 for normal and polysomy groups, p = 0.684 for polysomy and amplification groups, and p = 0.02 for normal and amplification groups. The conclusion is that normal and amplification groups are significantly different in term of extent. In term of intensity, the homogeneity of variance was found to not be fulfilled, thus instead of ANOVA, non-parametric Kruskal-Wallis test was used. It resulted in p = 0.116 meaning that we should retain the null hypothesis, namely that there is no significant difference between the three groups in term of intensity, however, the tendency for increased intensity from normal to amplification group can be established (Table 2).

## Discussion

As pan-TRK immunohistochemistry has a high negative predictive value, only pan-TRK immunopositive cases were investigated with *NTRK* probes to find *NTRK* gene alterations.

We studied 132 dedifferentiated liposarcoma cases after pan-TRK immunostaining by using *NTRK1/2/3* locus specific break-apart probe set. FISH was successfully performed in 65 of the 76 pan-TRK IHC preselected cases, and amplification patterns of one of the *NTRK* genes was seen in 18 cases (27.6%). A partial amplification pattern, meaning that only one of the two break apart FISH probes showed amplification was observed in 5 cases. The partial amplification pattern might be compatible with fusion of the involved genes, similar to cases described in dermatofibrosarcoma protuberans, with *COL1A1::PDGFB* or in case of *EWSR1::NFATC2* fusion (*EWSR1* pattern of FISH) in round cell sarcomas with *EWSR1*–non-ETS fusions [32]. Therefore, we performed NGS of one of these cases (case number: 112), but there was no *NTRK* fusion identified. Full transcriptome sequencing or whole genome sequencing might be needed to detect rare *NTRK* fusions with this type of FISH pattern. So far, there is only a limited literature on dedifferentiated liposarcomas harboring *NTRK* fusions. In a letter format, the authors reported two cases of dedifferentiated liposarcomas with *NTRK* fusions, though they admit that the functional significance was not clinically demonstrated” [33]. In a study trying to find *NTRK* fusion in solid tumors, one retroperitoneal liposarcoma showed double *NTRK3* fusions (*MORF4L1:NTRK3* and *PPFIA2:NTRK3*). Only 40 cases showed *NTRK* fusions among the investigated 10.194 patients with solid tumors (0.4%), and it was most frequently identified in soft tissue sarcomas (3.0%). Among soft tissue sarcomas, the fibrosarcoma subtype had an exceptionally high prevalence for *NTRK* fusions (12.7%) [34].

We found 18 cases with mutually exclusive amplification *NTRK* genomic loci. To our knowledge, the link between *NTRK* amplification and dedifferentiated liposarcoma was not investigated directly ever before. As 18/65 (27.6%) of the selected cases showed an amplification of in one of the *NTRK* genes, we further searched publicly available data derived from liposarcoma samples (both dedifferentiated and well-differentiated liposarcoma cases) from cBioPortal to estimate the frequency in an unbiased sample set. Amplification of the *NTRK* genes were observed 23/265 (8.6%) samples. This observed frequency is much lower than those observed in our study, however, here samples were analyzed without preselection for the presence of *NTRK* expression by IHC. Soft tissue sarcomas were screened for *NTRK* gene alterations other than fusions only in a few research [33–36]. *NTRK* amplifications were found in some tumors, like biliary tract cancers [37] and non-small cell lung cancer [38].

Unsurprisingly, as the focus of researches is still on fusions, the correlation of pan-TRK immunohistochemistry and *NTRK* amplification was not clarified before. Among 27 *NTRK* amplified tumors with various histology types only 4 showed positive pan-TRK immunostaining [39]. Two cases of soft tissue spindle cell tumors with *NTRK* fusion and co-occurring amplification as well showed positive pan-TRK immunoreaction [35]. Pan-TRK immunopositivity was seen in two cases of gastric cancers harboring *NTRK* amplifications [40].

There is no literature of partial *NTRK* amplification in tumors, and neither of polysomy in dedifferentiated liposarcoma at all, to our knowledge. However, polysomy and/or amplification of some genes (i.e., *ALK* and *HER2*) may indicate therapy against the tumors harboring these genes. In this way further investigation is needed whether it can be applicate in cases of DDLPSs with *NTRK* polysomy or amplification.

Viewing the *NTRK* gene family status by FISH, comparing it with pan-TRK immunohistochemistry, we can summarize that tumors with *NTRK* amplification are more likely to have a stronger and more diffuse pan-TRK immunoreaction, than those without *NTRK* rearrangement. Furthermore, cases with polysomy as well have a higher tendency to express a wider and more intense pan-TRK immunoreaction, but falling behind the *NTRK* amplified ones in that regard.

The relation between the gain of these genomic loci and increased expression of the gene regarding functional activity of the involved receptors requires further investigation.

## Data Availability

The datasets presented in this study can be found in online repositories. The names of the repository/repositories and accession number(s) can be found in the article/Supplementary Material.
